# Protective Efficacy of *Streptococcus Thermophilus* Against Acute Cadmium Toxicity in Mice

**Published:** 2018

**Authors:** Nanis G. Allam, Ehab Mostafa M Ali, Samya Shabanna, Elsayed Abd-Elrahman

**Affiliations:** a *Microbiology unit, Botany Department, Faculty of Science, Tanta University, Tanta, Egypt.*; b *Biochemistry unit, Chemistry Department, Faculty of Science, Tanta University, Tanta, Egypt.*

**Keywords:** Probiotics, Lactic acid bacteria, Streptococcus thermophiles, Detoxification, Cd toxicity

## Abstract

Cadmium (Cd) is a highly toxic heavy metal, wide occupational and an environmental pollutant, affecting human health. Probiotics especially lactic acid bacteria (LAB) have the capacity to bind, remove and to decrease tissue cadmium levels. The objective was to evaluate the potency of Cd binding capacity, antioxidative properties of probiotic bacteria against cadmium *in-vitro *and its probable detoxification effect against Cd-induced toxicity in mice. To asses this objective, resistance against cadmium and antioxidative properties (via DPPH radical scavenging and inhibition of lipid peroxidation) were estimated for thirteen probiotic bacteria. *Streptococcus thermophilus* was selected among investigated bacteria as it had the highest MIC against cadmium and remarkable antioxidant activities for treatment of Cd toxicity in Swiss albino mice by preventive and therapeutic protocols. Blood cadmium levels, reduced glutathione (GSH), malondialdehyde (MDA) and histopathological changes in the liver of mice were estimated at 6, 24 and 48 h post to acute Cd exposure (oral dose with 50 mg/kg body weight). On exposure to Cd a significant increase in blood Cd, MDA and reducing in GSH levels were observed. *S. thermophilus* offered a significant protective effect against Cd toxicity by decreasing the cadmium levels in blood and attenuation alterations in the levels of GSH and MDA and improved hepatic histopathological changes caused by Cd toxicity. These results indicated the protective action of *S. thermophilus* against acute cadmium toxicity as well as their beneficial health effects and suggested its use as a safe and efficacious nutritional dietary supplement to reduce cadmium toxicity.

## Introduction

Cadmium (Cd) is a heavy metal, environmental pollutant that is present as a contaminant in food, water, air and soil especially in developing countries (1). People mainly exposed to cadmium through fume inhalation, cadmium-nickel batteries, paint pigments, fertilizers, electroplating and the mining industries (2, 3). Cd is an extremely accumulative toxicant with long biological half-life (4). Liver and kidney are critical target organs to Cd exposure, because these organs contain most of the metallothionein (MT), a metal binding protein that plays an important role in protecting against Cd toxicity produced (5). Acute administration of Cd causes hepatotoxicity and nephrotoxicity following chronic exposure. It›s reported that the toxicity of Cd may be associated with the depletion of reduced glutathione (GSH), enhanced production of reactive oxygen species (ROS) and inhibition of antioxidant enzymes (6-8). It has been also reported that Cd induces free radical generation, resulting in oxidative degradation of lipids, proteins and DNA and enhancing various pathological conditions in humans and animals (9). The chelation therapy is the preferred medical treatment to reduce acute cadmium intoxication. Chelators such as monoisoamyl meso-2, 3-dimercaptosuccinate (MiADMS), and di- thiocarbamates are effective only if given a short time after cadmium exposure (10, 11) and cannot reduce the oxidative stress in the liver or brain (12). Antioxidants are another therapeutic possibility to alleviate cadmium induced oxidative stress, but several of them, including melatonin and α-lipoic acid, failed to reduce the cadmium limit in animal tissues (13, 14).

Probiotics are living microorganisms which confer beneficial effects on the hosts (15). Lactic acid bacteria (LAB) and bifidobacteria are the most common types of bacteria used as probiotics. LAB are non-pathogenic, safe bacteria that are used in the production of many fermented food products, they are a part of the microbial population of the digestive tract of healthy humans and animals, and are involved in their metabolism (16). Many scientific studies demonstrated a role of LAB in the protection of human and animal health due to their antimicrobial, immunomodulatory, anti-carcinogenic, anti- allergenic, anti-diarrheal, and antioxidant activities (17).

It’s reported that different LAB strains such as *Lactobacillus rhamnosus, L. plantarum, S. thermophilus, L. brevis* can bind and remove heavy metals *in-vitro* (18-24). Beside cadmium binding capacity LAB also reported to have antioxidative activities, which maybe another important advantage for cadmium toxicity protection (25, 26). On the basis of these special characteristics, daily LAB consumption could prove to be a protective dietary strategy for the populations exposed to Cd. Therefore, in this study, a probiotic bacterium strain with good cadmium binding capacity was selected for animal experiments. The effects on the cadmium limit reduction and oxidative stress alleviation in mice were assessed to investigate whether the cadmium binding capacity and anti-oxidative property of certain bacteria might play a potential role against cadmium toxicity.

## Experimental

*Chemicals and Reagents*

Cadmium chloride powder and other analytical laboratory chemicals and reagents were purchased from Sigma Chemical Company, USA. Kits used to measure the levels of MDA and GSH were purchased from Biodiagnostic Company, Giza, Egypt. De Man, Rogosa, and Sharpe (MRS) broth media and MRS agar were obtained from Oxoid Ltd., UK.

*Bacterial strains and culture*

*Streptococcus thermophilus *, *Streptococcus lactis* subsp*. Cremoris*, *lactobacillus casei, Lactobacillus delbrueckii* subsp*. bulgaricus, Lactobacillus delbrueckii* subsp. *bulgaricus* DMSZ 20081 T, *Lactobacillus fermentum* DSMZ 20049 , *Lactobacillus plantarum* DMSZ20079 T, *Lactobacillus rhamnosus*, *Bifidobacterium longum* subsp. *longum *DSMZ 200707, *Bifidobacterium bifidum* DSM 26082 ,* Lactobacillus rhamnosus* ATCC 7469 *, Lactobacillus reutri* DSM20016, *Lactobacillus acidophilus* DSMZ 20079 T were kindly provided from Faculty of Science, Tanta University and microbiological recourses center (Cairo MIRCEN), Faculty of Agriculture, Ain Shams University , Egypt. All strains were cultured in MRS broth at 37 °C for 24 h. To obtain living biomass for animal treatment, the cultured biomass was washed twice with ultra pure water, lyophilized with reconstituted skimmed milk as its protectant, and then stored at −20 °C. Colony counting was performed before animal experiments to ensure the survival of bacteria in the preparations for animal treatment.

*Determination of probiotic bacterial tolerance to Cd*

The Cd tolerance of each strain was determined by the minimum inhibitory concentration (MIC) approach. MICs of each bacterial strain were detected against Cd as cadmium chloride (CdCl_2_ .2½ H_2_O) separately. The concentration of Cd solution added was gradually increased (0.1 – 1 mM) by increasing amounts of metal salt added to the media. Each bacterial strain was inoculated on the surface of MRS agar plates supplemented with each concentration of metal solutions. The plates were incubated for 24 h at 37 ºC. The concentration of metal was increased till MICs was achieved as visualized by cessation of growth according to Washington and Sutter (27).

*Determination of antioxidant activity of probiotic bacteria*

*α,α-Diphenyl-β-Picrylhydrazyl (DPPH) scavenging assay*

The ability of probiotic bacteria to scavenge DPPH radicals was determined by a method of Molyneux (28). 0.1 mL of cell- free extracts (CFE) of probiotic bacteria (after 24, 48, 72 and 96 h incubation) in a test tube was mixed well with 3.9 mL of methanol and 1.0 mL of DPPH solution (0.025 g/L methanol; Sigma, USA). The mixture was kept at ambient temperature for 60 min in the dark prior to measurement of the absorbance at 515 nm. The mixture of DPPH and methanol was used as the blank sample. All measurements were done in triplicate. The scavenged DPPH was analyzed by measuring the decrease in absorbance at 515 nm. The scavenging ability was defined as follows equation (Eq. 1):

Eq. 1 DPPH scavenging % = (A_o_ – A_s_/A_o_) X 100

Where *A*_o_: is the absorbance of the blank. *A*_s__: _is the absorbance of sample at 515 nm.

*Lipid peroxidation inhibition assay*

The thiobarbituric acid (TBA) method was performed to measure the inhibition ability of the LAB strains on lipid peroxidation (29). FeCl_3_ was used to induce the liver homogenate peroxidation. 1 mL of liver homogenate (each 100 mL homogenate solution contains 1.0 g rat liver, 100 μL PBS buffer, 1mM FeCl3, 200 μL ascorbic acid and cell-free extracts of probiotic bacterial strains were mixed. The mixture was incubated at 37 °C for 60 min, then 1.0 mL of trichloroacetic (15%) and 1 mL of TBA (0.67%) was added and the mixture was heated up in boiled water for 15 min. The absorbance was recorded at 532 nm. The percentage of inhibition effect was calculated according to Eq. (2):

Eq. 2 The level of inhibition (%) on lipid peroxidation= [A1- A2/A0] × 100%

Where: A0 is the absorbance of the control (without sample), A1 is the absorbance of the sample addition, A2 is the absorbance without liver homogenate.

*Cadmium Binding capacity of S. thermophilus*

*S. thermophilus* was selected among investigated probiotic bacteria for further studies as *S. thermophilus* had the higher MIC level against cadmium and revealed remarkable antioxidative properties. The cadmium binding capacity of *S. thermophilus* was investigated according to Halttunen *et al.* (19). Bacterial culture was centrifuged at 7000 rpm for 15 min and washed twice to obtain the cell pellets. The cell pellets was resuspended in distilled water containing 5 mg/L cadmium ion as cadmium chloride (CdCl_2_ 2.½ H_2_O) to give a final bacterial concentration of 1 g/L on a dry weight basis. The pH of the suspension was immediately set to 6 using diluted NaOH or HNO_3_ and the samples were incubated for 1 h at 28 °C followed by a centrifugation at 7000 rpm for 15 min. After centrifugation, the residual cadmium content of the supernatant was measured by atomic absorption spectrophotometry (Spectr AA 220; Varian). The control was conducted in the cadmium-free distilled water. All the assays were performed in triplicate and average values were used for data analysis. 

*Transmission Electron Microscopy (TEM)*

After the metal binding experiment the bacterial pellets of the control and treated cultures were examined by TEM in order to identify the location of cadmium particles within the bacterial cells according to Halttunen *et al*. (21). The control and Cd treated cells were fixed with 5% glutaraldehyde (Merck, Darmstadt, Germany) in 0.16 M s-collidine buffer (pH 7.4) and dehydrated with series of ethanol. Dry pellets were embedded in epoxy resins (Glycidether 100, Merck) and cut into thin sections. Thin sections were viewed under JEM-1200EX transmission electron microscope (JEOL, Tokyo, Japan).

*Protective effects of S.thermophilus against acute Cd exposure*

*Animal*

Eighty adult male Swiss albino mice, weighing range from (25-30 g), were purchased from the animal house unit, National Research Centre, Giza, Egypt to be used throughout this work. The animals were housed in steel mesh cages and maintained for one week acclimatization period on commercial standard and pellet diet and drinking water *ad libitum. *The housing cycle was 12:12 hr light-dark cycle under controlled temperature (20-22 ºC). All the protocols of the present study were approved by the ethics committee of Tanta University, Egypt. 

*Experimental design*

Eighty mice were randomly divided into main four groups: negative (-ve) control (n = 10), positive (+ve) control (n = 10), prevention (P) and therapy (T) groups. In both prevention and therapy groups, mice were divided into three sub groups (n = 10 for each); one of them received *S. thermophilus* without exposed to Cd and other two subgroups were received *S. thermophilus* or skimmed milk (SM) and exposed to acute single dose of Cd. In prevention subgroups, mice received *S. thermophilus *at (1×10^9^ CFU) with 0.5 mL SM or 0.5 mL SM once daily for 7 days pre to acute cadmium exposure (50 mg/kg BW). While, in therapy subgroups, mice received *S. thermophilus *at (1×10^9^ CFU) with 0.5 mL SM or 0.5 mL SM 1 h post to acute cadmium exposure (50 mg/kg BW) at first day and continue to receive *S. thermophilus *till the end of experiment.

Mice were received SM, Cd and *S. thermophilus* orally via gavage; SM was served as a vehicle of *S*. *thermophilus* so doses of *S. thermophilus* were administered with 0.5 mL SM. Mice were fasted for 12 h before cadmium exposure. The dose of Cd was selected according to Zhai *et al.* (24) and Anderson *et al*. (30). During the time course of the experimental period, three rats of each group were randomly chosen and sacrificed after 12, 24 and 48 h after Cd exposure in order to measure the level of Cd in blood, estimation of Malondialdehyde (MDA) and glutathione reduced (GSH) in liver tissue. Also, the histopathological changes in liver were detected.

**Table 1:** DPPH radical scavenging activity of probiotic bacteria

**probiotic bacteria**	**DPPH%** **Incubation period (h)**			
	**24**	**48**	**72**	**96**
*B. bifidum* DSM 26082	85.56 ±0.25^A a^	86.06±0.35^A a^	83.43± 0.25^B a^	85.63±0.20^A a^
*L. reutri* DSM20016	86.63± 0.20 ^A b^	87.3±0.35 ^B bd^	82.31± 0.40 ^C b^	88.8±0.75 ^D b^
*L.rhamnosus* ATCC 7469	88.93±0.55 ^A c^	87.13±0.45 ^B b^	90.41±0.27^ C^^ ci^	90.21± 0.37 ^C fi^
*L.plantarum* DSMZ 2017	90.36±0.25^ A d^	87.63±0.45^B bd^	87.50±0.40^B d^	86.73±0.35^C d^
*B.longum* DSMZ 200707	86.01±1.01^A ab^	83.51±0.22^B d^	79.36±0.28^C^^ e^	82.46±0.32^D e^
*L.lactis*	89.8± 0.27^A^^ d^	87.30±0.16^B^^ b^	89.93±0.25^A ij^	89.76±0.15^A cf^
*L.rhamnosus*	84.41±0.22^A e^	88.65± 0.32^B c^	90.67±0.15^C cj^	89.76±0.20^D f^
*L.casei*	82.66±0.25^A f^	84.41±1.45^B e^	84.45±0.23^B f^	84.36±0.25^B g^
*S.thermophilus*	87.58±0.37^A g^	89.31±0.18^B f^	88.43±0.27^C^^ g^	87.41±0.18^A h^
*L.bulgaricus*	84.50±0.25^A e^	84.90±0.26^B e^	89.37±0.29^C^^ h^	84.12± 0.14^A g^
*L.bulgaricus* DSMZ 20081	90.55±0.22^A d^	87.61± 0.26^B bd^	90.15±0.32^A j^	90.63±0.23^A i^
*L.acidophilus* DSMZ 20079 T	83.34±0.21^A h^	88.41±0.22^B c^	89.55±0.21^C hij^	84.20±0.36^D g^
*L.fermentum *DSMZ 20049	89.11±0.31^A c^	87.53±0.23^B bd^	86.71±0.28^C k^	87.96±0.35^B h^

All values are expressed as mean of three replica ± standard deviation. Different capital letters indicate statistically significant differences at *p *< 0.05 within each row comparisons, different small letters indicate statistically differences at *p *< 0.05 within the same column comparisons

**Table 2:** Lipid peroxidation inhibition ability of probiotic bacteria

**Probiotic bacteria**	**Inhibition of lipid peroxidation%**
*B. bifidum* DSM 26082	50.16±0.87^a^
*L. reutri* DSM20016	70.35±1.21^b^
*L.rhamnosus* ATCC 7469	61.80±1.40^c^
*L.plantarum* DSMZ 2017	78.52±0.56^d^
*B.longum* DSMZ 200707	65.72±1.07^e^
*L.lactis*	72.53±1.27^f^
*L.rhamnosus*	75.33±0.83^g^
*L.casei*	70.53±0.74^b^
*S.thermophilus*	73.28±1.10^f^
*L.bulgaricus*	71.56±0.27^bf^
*L.bulgaricus* DSMZ 20081	79.32±0.72^d^
*L.acidophilus* DSMZ 20079 T	75.12±0.93^g^
*L.fermentum *DSMZ 20049	70.17±0.80^b^

All values are expressed as mean of three replica ± standard deviation. Different small letters indicate statistically differences at *p *< 0.05.

**Table 3:** Effects of *S. thermophilus* on concentrations of Cd in blood of mice

**Groups**	**Cd (µg/L)**		
	6 h	24 h	48 h
-ve control	0	0	0
+ve control (Cd)	64.00±1.15 ^A e^	28.70±0.26^ B c^	11.76±0.51^C c^
**Prevention**			
*S.thermophilus*	0	0	0
Cd + SM	57.17±1.25^A c^	32.20±1.24^B c^	11.80±0.36^C c^
*S.thermophilus* + Cd	22.70±1.45^A a^	10.83±0.41^B a^	3.43±0.25^C a^
**Therapy**			
*S.thermophilus*	0	0	0
Cd + SM	63.36±1.33^A e^	28.03±1.51^B c^	11.67±0.90^C c^
*S.thermophilus* + Cd	39.30±1.20^A d^	17.46±1.23^B e^	6.13±0.45^C e^

All values are expressed as mean of three replica ± standard deviation. Different capital letters indicate statistically significant differences at *p *< 0.05 within each row comparisons, different small letters indicate statistically differences at *p *< 0.05 within the same column comparisons.

**Table 4:** Effects of *S. thermophilus* on cadmium-induced alterations of the activities of GSH and MDA levels in the livers of mice

**Groups**	**MDA nmol/ g tissue**			**GSH nmol/ g tissue**			
	**6 h**	**24 h**	**48 h**	**6 h**	**24 h**	**48 h**	
-ve control	126.40±2.94^A d^	123.00±4.00^A b^	123.00±4.03^A d^	13.26±0.63^A b^	12.98±0.29^A b^	13.39±0.38^A b^	
+ve control (Cd)	351.33± 5.50^A c^	290.67±5.03^B e^	211.33±6.65^C c^	3.64±0.16^A e^	5.62±0.30^B e^	8.57±0.39^ C c^	
**prevention**							
*S.thermophilus*	111.50±1.80^A b^	117.00±3.00^A b^	114.00±6.24^A b^	14.22±0.31^A b^	14.31±0.34^A b^	14.38±0.26^A b^	
Cd + SM	348.66±5.13^A c^	278.66±4.04^B c^	205.00±3.60^C c^	3.74±0.12^A c^	5.77±0.25^B c^	8.35±0.23^C c^	
*S.thermophilus* + Cd	242.00±4.20^A a^	193.00± 4.15^B a^	148.66±2.51^C a^	6.64±0.24^A a^	8.95± 0.20^B a^	11.03±0.18^C a^	
**therapy**							
*S.thermophilus*	129.66±4.27^A d^	126.66±3.38^A f^	126.6±4.04^A d^	12.93±0.25^A b^	13.32±0.28 ^A b^		12.51±0.11^A b^
Cd + SM	351.66±4.16^A c^	295.67±3.51^B e^	207.66±3.05^C c^	3.69±0.17^A e^	5.81±0.05^ B e^		8.56±0.45^C c^
*S.thermophilus* + Cd	272.33±5.03^A e^	212.60±4.08^B d^	176.66±4.04^C e^	6.033±0.13^A d^	8.32±0.16 ^B d^		10.34±0.10^C d^

Values are mean ± SD values with ten mice in each group .different capital letters indicate statistically significant differences at *p *< 0.05 within each row comparisons, different small letters indicate statistically differences at *p *< 0.05 within the same column comparisons. T: therapy group; P: prevention group; LAB: *S.thermophilus*

**Figure 1:**
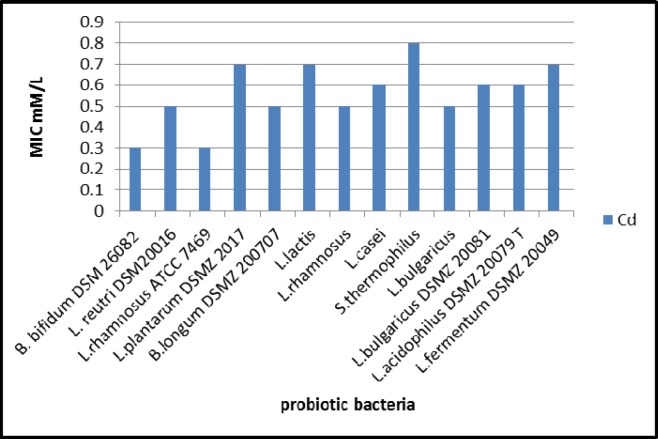
The minimum inhibitory concentration (MIC, mM) of cadmium against the tested probiotic bacteria.

**Figure 2A:**
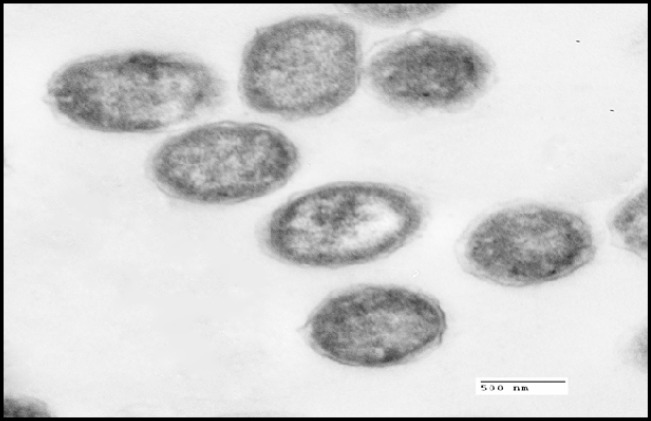
Transmission electron micrographs of *S. thermophilus* without addition of cadmium (control).

**Figure 2B:**
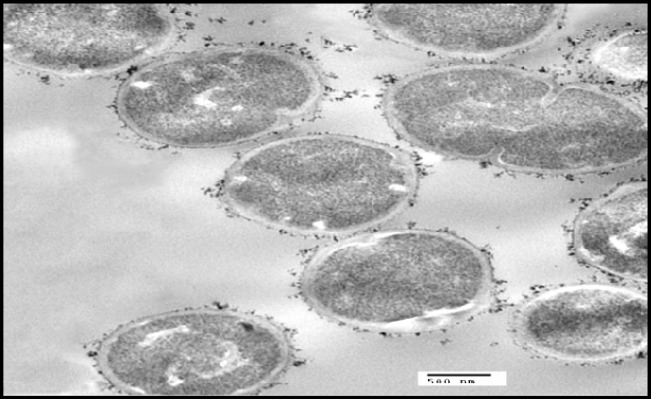
Transmission electron micrographs of *S. thermophilus* with addition of cadmium, Particles of cadmium were clearly visible on the surface of the bacterial cell

**Figure 3A:**
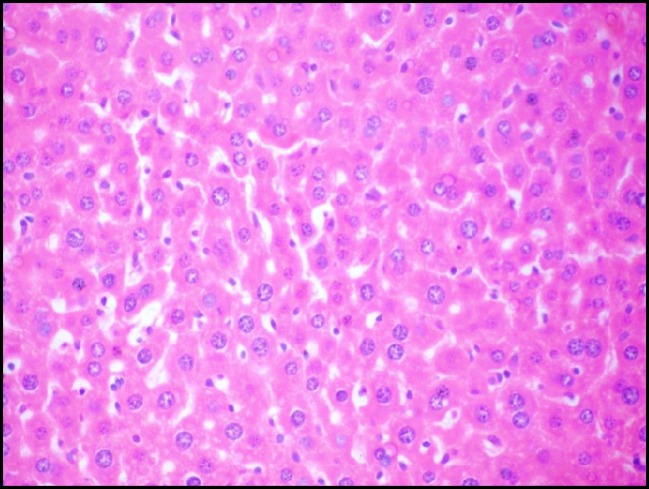
Photomicrograph of liver of mice (H&E, 400×) from -ve control group showing the normal hepatic histological structure

**Figure 3B:**
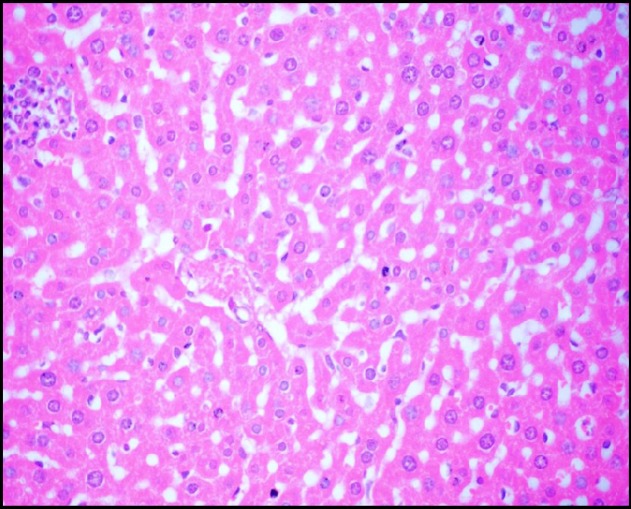
Photomicrograph of liver of mice (H&E, 400×) from + ve control (Cd) group showing cytoplasmic vacuolization and necrosis of hepatocytes

**Figure 3C:**
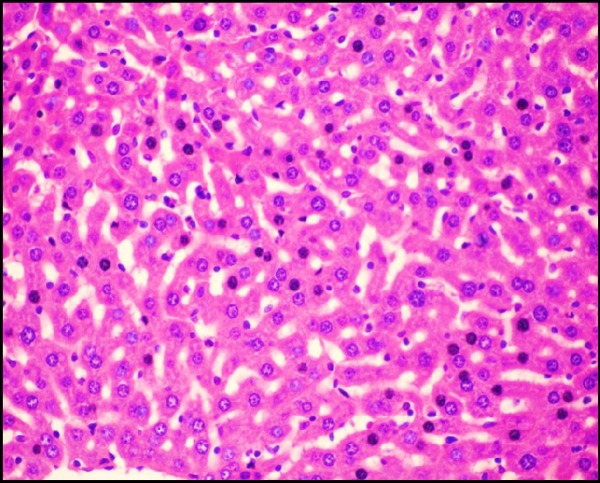
Photomicrograph of liver of mice (H&E, 400×) from *S.thermophilus* + Cd prevention group showing No necrosis and reduction of cytoplasmic vacuolization.

*Samples preparation*

Animals were anesthetized with ether before blood collection from the eye (31). Blood was collected into heparinized tubes and mixed well to prevent clot formation; tubes were marked with numbers and were stored refrigerated at (2-4 ºC) until assayed for blood cadmium levels by using atomic absorption spectrophotometer according to method of Memon *et al.* (32).

Livers were removed, cleaned, and stored at -80 °C until the biochemical assays that include: Malondialdehyde (MDA) and glutathione decreased (GSH). Parts of Livers from each group were fixed for 48 h in 10% formalin saline for light microscopy examination.

*Determination of cadmium level in blood of mice*

Blood samples were digested in concentrated HNO_3_ according to Memon *et al*. (32). The cadmium concentration was determined by a flame or graphite furnace atomic absorption spectrophotometer (Spectr AAS or AA; Varian). The cadmium in blood was expressed in µg/L.

*Determination of hepatic MDA and GSH*

The levels of MDA and GSH were measured according to the recommendations of the manufacturer, using an assay kit purchased from Biodiagnostic Company, Giza, Egypt.

*Histopathological studies*

Parts of livers were fixed for 48 h in 10% formalin saline. Tissues were embedded in paraffin and sectioned at 5 µm thickness using a rotary microtome. Sections were stained with hematoxylin-eosin (H&E) for light microscopy examination (33).

*Statistical analysis*

Data were expressed as the mean standard deviation (SD) for each group. Differences between groups were analysed using one-way and two-way analysis of variance (ANOVA). *P *value of ≤ 0.05 was considered to be statistically significant.

## Results

*Cadmium tolerance of probiotic bacteria*

The growth of all investigated bacteria was decreased gradually by increasing the concentration of cadmium on the MRS media. The results as illustrated in Figure 1 revealed that *S. thermophilus* was the most tolerant strain to Cd as it recorded the highest MIC value among other investigated bacteria.

*Antioxidant activities of probiotic bacteria in-vitro*

*DPPH radical scavenging*

DPPH radical scavenging activity of cell free supernatants of probiotic bacteria were assayed after 24, 48, 72 and 96 h of incubation periods as shown in Table 1, the tested bacteria revealed little difference in DPPH scavenging activity. The highest activity was recorded by *L. rhamnosus* after 72 h of incubation with percentage of 90.67 %,* L. plantarum* after 24 h of incubation with percentage of 90.3 % and *S.thermophilus* after 48 h of incubation with percentage of 89.31%.While, *B. longum* DSMZ 200707 had the lowest activity with percentage of 79.36% after 72 h of incubation.

*Inhibition of lipid peroxidation in rat liver homogenate*

As shown in Table 2, the ability of different probiotic bacteria to inhibit lipid peroxidation was ranging from 50% to 79% approximately. *L. plantarum *DSMZ 2017 and *L. bulgaricus* DSMZ 20081 had the highest inhibition percentage (78.52 ± 0.56 and 79.32 ± 0.72 respectively) without significant differences. While, *B. bifidum* DSM 26082 had the lowest inhibition percentages (50.16 ± 0.87).* S. thermophilus* had inhibition percentage of 73.28 ± 1.10. Based on the previous recorded results,* S. thermophilus* was selected for ingoing experiments as it revealed strong antioxidant activity (inhibiting MDA with 73.28% and scavenging DPPH with 89.31% after 48 h) and tolerated Cd till 0.8 mM.

*Cadmium Binding capacity of S. thermophilus*

Cadmium binding capacity of *S. thermophilus *was recorded with biosorption of 3.527 mg/g dry biomass and 70.54% cadmium removal from aqueous solution. This led to a hypothesis that *S. thermophilus* might be able to reduce cadmium toxicity in mice.

*TEM examination*

*S. thermophilus* was analyzed for its ability to bind and sequester cadmium. Particles of Cd were clearly visible on the surface of the bacterial cell as illustrated in Figure 2B whereas no Cd was visible on control micrograph (Figure 2A). The obtained results indicate that binding of Cd occurred at the surface of *S. thermophilus* cell without visible uptake or precipitation inside the cell.

*Protective effects of S. thermophilus against acute Cd exposure*

*Cadmium concentration in blood of mice*

Cadmium exposure model in mice was established to determine the effects of *S. thermophilus* on the reduction of Cd toxicity. Blood Cd concentrations in the -ve control and *S. thermophilus*- only groups were represented as zero because it was too low to be detected. The cadmium concentration was significantly increased in the first 6 h after oral exposure to Cd and decreased to its lowest level after 48 h as shown in Table 3. contrarily, Cd-treated groups with *S. thermophilus* revealed a significantly reduction in blood cadmium levels after 6 h, 24 h and 48 h *(p *≤ 0.05) in both prevention and therapy groups post to Cd exposure. The protection was more prominent in prevention group than that in therapy group.

*Hepatic MDA and GSH of mice*

The results in Table 4 demonstrated changes of hepatic GSH and MDA concentrations in the liver of mice at 6, 24, and 48 h post to Cd exposure. GSH levels were significantly decreased in both prevention and therapy of Cd-treated groups and were accompanied with a significant increase in the levels of MDA (*p *≤ 0.05). The changes in GSH and MDA levels were noticed after 6 h of Cd exposure and still significantly different in comparison to control groups even after 48 h.

The effects of *S.thermophilus *on the alterations of GSH and MDA were significantly noticed in prevention group than therapy one.

*Effect of S. thermophilus on Cd- induced changes on histological structure of mice livers*

Liver tissue of mice from -ve control group and the mice that received *S.thermophilus *only showed normal hepatic histological structure (Figure 3A) as shown by light microscope. While, acute cadmium exposure caused marked damage of hepatocytes in the form of chromatin condensation, cytoplasmic vacuolization and necrosis of hepatocytes (Figure 3B). *S.thermophilus* treatment markedly alleviated such cadmium-induced hepatic injury (Figure 3C) of mice liver of prevention group.

## Discussion

This study provides evidence that *S.thermophilus* has antioxidant properties, good Cd binding capacity and could offer a significant treatment against environmental Cd contamination and detoxification effect against Cd toxicity in mice.

The antioxidative ability might be one of the main mechanisms of probiotic bacteria to alleviate cadmium toxicity. In this study all tested LAB showed remarkable DPPH scavenging activity and inhibition of lipid peroxidation *in-vitro*. The antioxidant properties of LAB had been reported by many researches (25, 34, 35). The majority of milk bacteria show antioxidant behaviour, eliminating the excess oxygen free radicals and producing superoxide dismutase, or glutathione (36). The yoghurt bacteria *Lactobacillus delbrueckii* and *Streptococcus thermophilus* inhibited peroxidation of lipids through scavenging the reactive oxygen radicals, such as hydroxyl radical, or hydrogen peroxide (37). Hence, we hypothesized that treatment with *S.thermophilus* can offer protection against Cd-induced oxidative stress.

In the present study, the tested probiotic bacteria showed high tolerance levels against cadmium ions on MRS media with MICs up to 0.8 mM by *S. thermophilus*. Moreover, the MICs of different LAB against Cd were varied in this study; such differences may be due to the specific properties, such as structure of the bacterial cell, functional groups on the cell surface, and surface area, depending on the bacterial division, genera, and species (38). The mechanisms of cadmium binding by different bacteria may be complex. Ion exchange could be possibly responsible for cadmium binding capacity (39, 19). Also, anionic groups on the cell surface play important roles in metal binding. The surface of LAB cell wall is composed of a thick peptidoglycan layer with proteins, teichoic acids, and other extracellular coatings such as capsular polysaccharides (40). The presence of functional groups on the cell surface of LAB including hydroxyl and phosphate groups, offers specific roles in binding of cadmium (21). This can explain the cadmium binding capacity of *S.thermophilus *which removed approximately 70 % from aqueous solution in short time (1 h) In the same connection, Zhai *et al*. (24) tested nine LAB strains for Cd binding capacity and found that the levels of cadmium removal were ranged between 29 % - 77 %. TEM micrographs revealed that particles of Cd were clearly visible on the surface of the bacterial cell, indicating that Cd binding occurred at the surface of *S. thermophilus *cell. In confirm, a previous *in-vitro* study showed that TEM images revealed the electron-dense layer that represents cadmium particles throughout the cell wall only, with no intracellular accumulation (41). 

The significant ability of *S. thermophilus* to reduce cadmium levels in blood of mice after acute exposure, may be attributed to fast and efficient cadmium binding and removal capacities of* S. thermophilus*. We supposed that *S. thermophilus* can decrease absorption of cadmium in the intestine at an early phase of cadmium exposure and prevent the transportation of high level of Cd into the body. A similar mechanism was reported for *L. plantarum* CCFM8610 which could alleviate cadmium toxicity in mice by decreasing intestinal absorption of cadmium and increasing the faecal cadmium excretion after acute oral exposure (24).

Oxidative damage resulted from generation of reactive oxygen species (ROS) have been established as a mechanism of cadmium toxicity. Interaction of ROS with the cellular macromolecules causes lipid peroxidation, changing intracellular glutathione levels, DNA damage and membrane protein degradation (4). Thus leading to the necrosis of hepatocytes (42); this may be the reason for the hepatic damage observed in the histopathological examination of liver section of +ve control group (Cd group). 

Thiol groups especially GSH play an important role in the intracellular protection against toxic compounds and also in the detoxification and excretion of cadmium (43, 44). The obtained data revealed that the significant increase in the level of MDA accompanied by significant decrease in GSH level in the liver after acute cadmium exposure. The large amount of ROS formation due to cadmium exposure can lead to the depletion of antioxidant mechanisms, causing the exhaustion of antioxidant enzymes, reducing their activities and inhibiting defensive ability of liver cells (45, 46). The present study revealed that treatment with *S. thermophilus *alone caused slight positive changes in GSH and MDA levels; this may be attributed to the antioxidative properties of *S. thermophilus*. The reduction in MDA levels and the increase in GSH levels indicated that *S. thermophilus* had a significant ability to protect the liver against cadmium-induced oxidative damage in both prevention and therapy groups. We supposed that the treatment ability of *S. thermophilus* in preventive and therapeutic protocols may be due to the initial intestinal cadmium removal via its effective Cd binding ability beside the antioxidative properties.

Our experimental data showed that the reduction in MDA levels and the induction in GSH levels started 24 and 48 h after acute cadmium exposure. In confirm, a previous study showed that hepatic GSH levels return to normal after 24 and 48 h of acute Cd exposure, through a *de novo* GSH synthesis and Cd elimination (47).

The present results revealed that *S. thermophilus* treatment in the prevention group offered protection against cadmium intoxication more than in the therapy group. This may be due to administration of *S*.* thermophilus* for 7 days before Cd exposure which provided intestinal colonization and biomass of *S. thermophilus* ready to protect the body against Cd from the first moment of intoxication. 

## Conclusion

In conclusion, this study showed that *S. thermophilus* had protective effects against acute Cd toxicity in mice. This strain had anti-oxidative capacity, good Cd binding ability and could offer a significant protection against acute Cd toxicity in mice by decreasing blood cadmium concentrations, alleviating lipid peroxidation and hepatic oxidative stress and ameliorating hepatic histopathological changes. *S. thermophilus* can be used as a supplementary ingredient to provide a dietary strategy in the treatment and prevention of cadmium toxicity especially in industrial regions in developing countries. Further studies on the binding and anti-oxidative mechanisms of *S. thermophilus* *in-vivo* are recommended.
